# Comparison of the Sensitivity of QuantiFERON-TB Gold In-Tube and T-SPOT.*TB* According to Patient Age

**DOI:** 10.1371/journal.pone.0156917

**Published:** 2016-06-03

**Authors:** Won Bae, Kyoung Un Park, Eun Young Song, Se Joong Kim, Yeon Joo Lee, Jong Sun Park, Young-Jae Cho, Ho Il Yoon, Jae-Joon Yim, Choon-Taek Lee, Jae Ho Lee

**Affiliations:** 1 Department of Internal Medicine, Seoul National University College of Medicine, Seoul, Republic of Korea; 2 Division of Pulmonary and Critical Care Medicine, Department of Internal Medicine, Seoul National University Bundang Hospital, Seongnam-si, Gyeonggi-do, Republic of Korea; 3 Department of Laboratory Medicine, Seoul National University Bundang Hospital, Seongnam, Republic of Korea; 4 Department of Laboratory Medicine, Seoul National University College of Medicine, Seoul, Republic of Korea; 5 Division of Pulmonary and Critical Care Medicine, Department of Internal Medicine, Seoul National University Hospital, Seoul, Republic of Korea; University of Texas Health Northeast, UNITED STATES

## Abstract

Currently, there are two types of interferon-gamma release assays (IGRAs) in use for the detection of tuberculosis (TB) infection, the QuantiFERON-TB Gold In-Tube test (GFT-GIT) and T-SPOT.*TB*. Owing to contradictory reports regarding whether the results of these IGRAs are affected by the age of the patient, we aimed to determine if these two tests have age-related differences in sensitivity. We retrospectively reviewed the medical records of diagnosed TB patients who were tested using either QFT-GIT or T-SPOT.*TB* from February 2008 to December 2013. The positivity of the two tests was analyzed and compared with true TB infection, which was defined as active TB based on either a positive *Mycobacterium* culture or a positive TB polymerase chain reaction. The QFT-GIT group included 192 TB patients, and the T-SPOT.*TB* group included 212 TB patients. Of the patients with pulmonary TB, 76 (39.6%) were in the QFT-GIT group and 143 (67.5%) in the T-SPOT.*TB* group. The overall sensitivity was 80.2% for QFT-GIT and 91.0% for T.SPOT.*TB*. The sensitivities of QFT-GIT and T-SPOT.*TB* according to age group were as follows: <29 years, 93.3% and 96.7%; 30–49 years, 86.5% and 94.7%; 50–69 years, 76.8% and 87.5%; and >70 years, 68.3% and 85.7%, respectively. The trend of age-related changes in sensitivity was significant for both QFT-GIT (*p* = 0.004) and T.SPOT.*TB* (*p* = 0.039). However, only QFT-GIT was significantly related to age in the multivariate analysis. QFT-GIT, but not T-SPOT.*TB*, was significantly affected by patient age.

## Introduction

Although the incidence of and mortality due to tuberculosis (TB) is slowly decreasing worldwide, in 2013, approximately 9.0 million new cases of TB were reported, and annually, there are approximately 1.5 million deaths due to TB infection worldwide [1]. TB is a very prevalent infectious disease, and early and accurate diagnosis is essential to control its spread. The tuberculin skin test (TST) has been conventionally used as the standard diagnostic test for TB infection. However, it has a high false positive rate in patients who were vaccinated with bacille de Calmette-Guerin (BCG) or were infected with nontuberculous mycobacteria [2]. The interferon-gamma release assay (IGRA) was recently developed to overcome the limitations of the TST, and it is expected to be more useful for the diagnosis of TB infection [3, 4]. There are currently two types of commercial IGRAs available, the QuantiFERON-TB Gold In-Tube test (QFT-GIT) and the T-SPOT.*TB* blood test. Both tests are approved by the Food and Drug Administration as indirect tests for TB infection (including active disease) when used in combination with other medical and diagnostic evaluations [5]. QFT-GIT measures the concentration of interferon-gamma (IFN-γ) via an enzyme-linked immunosorbent assay (ELISA), whereas T-SPOT.*TB* measures the number of IFN-γ-secreting T cells via an enzyme-linked immunospot (ELISPOT) assay. The T-SPOT.*TB* method uses separate mixtures of ESAT-6 and CFP-10 synthetic peptides as *Mycobacterium tuberculosis*-specific antigens, whereas QFT-GIT uses a single mixture of synthetic ESAT-6, CFP-10, and TB7.7 peptides. Since aging leads to a decline in the strength of immune responses [6, 7], older individuals tend to be more susceptible to TB [8]. There is concern that this decline of immune responsiveness may decrease the sensitivity of IGRAs in aged populations [9, 10]. However, relatively few studies have examined the influence of age on IGRAs [9–16]. Most of these studies were performed with QTF-GIT alone, and the study results were contradictory [10, 12, 13]. In this study, we aimed to investigate the impact of patient age on the sensitivity of the two commercial IGRAs.

## Materials and Methods

In this study, the electronic medical records of patients who were evaluated by an IGRA and were finally diagnosed with active pulmonary or extrapulmonary TB at Seoul National University Hospital or Seoul National University Bundang Hospital from February 2008 to December 2013 were retrospectively analyzed. The Institutional Review Board (IRB) of Seoul National University Bundang Hospital, South Korea, and the IRB of Seoul National University Hospital, South Korea, approved the study protocol and waived the need for informed consent because no patients were at risk. Patient records were anonymized and de-identified prior to analysis.

Active TB was defined as follows: (1) culture-positive for mycobacteria; (2) the presence of caseating granulomas, together with a positive TB polymerase chain reaction (PCR) in tissue specimens obtained by biopsy or surgery; or (3) a positive MTB PCR in sputum, bronchial washing, or abscess aspiration samples. We reviewed the laboratory results for white blood cell (WBC) counts, percentage of lymphocytes, absolute lymphocyte count, and the levels of serum protein, serum albumin, and C-reactive protein (CRP). The patients were divided into four groups according to age: <29 years, 30 to 49 years, 50 to 69 years, and >70 years of age. Immunocompromised patients were defined as those who were infected with human immunodeficiency virus (HIV), had a history of organ transplantation, were on steroids or other immunosuppressive agents, had received cytotoxic chemotherapy, or had liver cirrhosis Child-Pugh class C, end-stage renal disease on hemodialysis, or diabetes mellitus (DM). Results that were indeterminate or negative were considered negative in the sensitivity analysis. All IGRA testing was performed before the patients were prescribed anti-TB medications.

### QuantiFERON-TB Gold In-Tube

Venous blood was collected into three heparinized tubes that were designated as follows: an antigen tube that contained TB-specific stimulating antigens (ESAT-6, CFP-10, and TB7.7), a mitogen (positive control) tube, and an antigen-free negative (nil) control tube. After 16–24 h of incubation at 37°C, the IFN-γ concentration was measured using an ELISA kit (Cellestis, a QIAGEN Co.). The test was performed and the results were interpreted according to the manufacturer’s guidelines [17].

### T-SPOT.*TB*

The T-SPOT.*TB* test was carried out according to the manufacturer’s recommendations [18]. Peripheral blood mononuclear cells (PBMCs) were isolated from a whole blood sample, washed, and counted. Then, the PBMCs were incubated with antigens to stimulate INF-γ secretion by the T cells. Secreted cytokine was captured by specific antibodies on the surface of the membrane, and unwanted materials were removed by washing. The spots were read using an ELISPOT plate reader (AID-GmbH, Straßberg, Germany). The results were interpreted as indeterminate if there were <20 spot-forming cells (SFCs) in the positive control or >10 SFCs in the negative well. Results were interpreted as positive under either of the following two conditions: 1) for a nil control with 0–5 SFCs, the number of SFCs in the ESAT-6 or CFP-10 wells minus the number of SFCs in the nil control >6; or 2) for a nil control with >5 SFCs, there were more than twice as many SFCs in the ESAT-6 or CFP-10 well as in the nil control. T-SPOT.TB was run within 8 hours, and the T-cell Xtend reagent was used to extend the storage time of the sample up to 32 h and to achieve accuracy equivalent to the standard T-SPOT.TB, which was not used in our institution [19–21].

### Statistical analysis

Data obtained from medical records were entered and analyzed using SPSS version 21 (SPSS Inc., Chicago, IL, USA). The sensitivity of each IGRA among the different age groups was compared using binary logistic regression and linear-by-linear association. Comparisons of continuous variables including WBC and lymphocyte counts, CRP, serum protein, and serum albumin levels, across age groups were performed using one-way analysis of variance (ANOVA) and post-hoc analysis. The effect of each factor on the sensitivity of each IGRA was analyzed by logistic regression adjusting for age group. A factor was considered to influence IGRA sensitivity when the age group was adjusted by a certain variable or some variables and the sensitivity of the IGRA according to age group was statistically insignificant. A *p* value less than 0.05 was considered significant.

## Results

### Sensitivity of IGRAs

The demographics and characteristics of the study population are presented in Table 1. The overall test sensitivity was 80.2% and 91.0% for QFT-GIT and T-SPOT.*TB*, respectively (Table 2). The sensitivity of GFT-GIT according to patient age group was as follows: <29 years, 93.3% (28/30); 30–49 years, 86.5% (45/52); 50–69 years, 73.8% (53/69); and >70 years, 68.3% (28/41). This trend of declining sensitivity with increasing age was linear and statistically significant (OR 0.555, 95% confidence interval [CI] 0.371–0.829, *p* = 0.004; Table 3), and was further confirmed by a linear-by-linear association (*p* = 0.003).

**Table 1:** Description of subjects in the two IGRA groups: QFT-GIT, T-SPOT.*TB*

Variable		QFT-GIT (N = 192)	T-SPOT.*TB* (N = 212)
Sex (M:F)		97: 95	104: 108
Age			
	≤29 years	30 (15.6)	30 (14.2)
	30–49 years	52 (27.1)	76 (35.8)
	50–69 years	69 (35.9)	64 (30.2)
	≥70 years	41 (21.4)	42 (19.8)
TB infection lesion			
	Pulmonary TB	76 (39.6)	143 (67.5)
	Both TB	34 (17.7)	31 (14.6)
	Extrapulmonary TB	82 (42.7)	38 (17.9)
Immunocompromised patients*		61 (31.7)	42 (19.8)
	HIV (+)	4 (2.1)	0
	Solid cancer on anticancer chemotherapy	5 (2.6)	4 (1.9)
	Hematologic malignancy	8 (4.2)	3 (1.4)
	End-stage renal disease	5 (2.6)	2 (0.9)
	Liver cirrhosis, Child-Pugh class C	0	1 (0.5)
	Organ transplantation	6 (3.1)	0
	Receiving immunosuppressant drugs	12 (6.3)	5 (2.4)
	Diabetes mellitus	29 (15.1)	30 (14.2)

Values are given as No. (%), unless otherwise indicated.

*Subjects may haver one or more underling disease.

**Table 2:** Results of the two IGRAs.

Result of IGRA	All subjects		Immunocompromised subjects	
	QFT-GIT (n = 192)	T-SPOT.*TB* (n = 212)	QFT-GIT (n = 61)	T-SPOT.*TB* (n = 42)
Positive	154 (80.2)	193 (91.0)	45 (73.8)	33 (78.6)
Negative	23 (12)	15 (7.1)	8 (13.1)	8 (19.0)
Indeterminate	15 (7.8)	4 (1.9)	8 (13.1)	1 (2.4)

Values are given as No. (%), unless otherwise indicated.

**Table 3:** Sensitivity of IGRAs across age group.

Test	Sensitivity					OR	95% CI	*p-value*
	≤29 years	30~49 years	50~69 years	≥70 years	Overall			
QFT-GIT (n = 192)	93.3%	86.5%	76.8%	68.3%	80.2%	0.555	0.371–0.829	.004
T-SPOT.*TB* (n = 212)	96.7%	94.7%	87.5%	85.7%	91.0%	0.579	0.345–0.974	.039

Trend of sensitivity was analyzed by binary logistic regression.

OR = odds ratio

The sensitivity of T-SPOT.*TB* according to patient age group was as follows: <29 years, 96.7% (29/30); 30–49 years, 94.7% (72/76); 50–69 years, 87.5% (56/64); and >70 years, 85.7% (36/42). Similar to QFT-GIT, the trend of declining sensitivity with increasing age was statistically significant (OR 0.579, 95% CI 0.345–0.974, *p* = 0.039; Table 3) and was corroborated by a linear-by-linear association (*p* = 0.036).

In terms of classification by infected TB lesion site, the sensitivity of QFT-GIT according to age group decreased in patients with either pulmonary or extrapulmonary TB. With T-SPOT.*TB*, the sensitivity according to age group decreased in pulmonary TB patients only, whereas patients with both pulmonary and extrapulmonary TB lesions had 100% of sensitivity in each age group.

### Laboratory data according to age

We analyzed the data for WBC counts, percentage of lymphocytes, absolute lymphocyte counts, and the levels of serum protein, serum albumin, and CRP. For QFT-GIT, the mean absolute lymphocyte count and levels of serum protein and albumin were significantly different in certain age groups by one-way ANOVA. For T-SPOT.*TB*, one-way ANOVA analysis determined that the mean percentage of lymphocytes, absolute lymphocyte count, and levels of serum protein and serum albumin were significantly different in certain age groups (Table 4).

**Table 4:** Other laboratory data of subjects according to age group (WBC, percent and absolute lymphocyte count, CRP, serum protein, serum albumin).

	QFT-GIT					T-SPOT.*TB*				
	≤29 years	30~49 years	50~69 years	≥70 years	*p-value*	≤29 years	30~49 years	50~69 years	≥70 years	*p-value*
WBC	6784 (±2148)^a^	6816 (±2995)^a^	6769 (±3023)^a^	6864 (±3267)^a^	.999	6350 (±1850)^a^	7097 (±2622)^a^	6980 (±2591)^a^	6853 (±2672)^a^	.587
Lymph-percent	21.7 (±7.5)^a^	23.3 (±12.6)^a^	20.9 (±11.2)^a^	18.3 (±14.6)^a^	.271	24.5 (±9.9)^a,b^	24.7 (±9.4)^a,b^	27.4 (±14.3)^b^	18.2 (±8.4)^a^	.001
Abs-lymph-count	1412 (±579)^a,b^	1465 (±702)^a^	1268 (±688)^a,b^	1039 (±546)^b^	.014	1474 (±553)^a,b^	1581 (±802)^b^	1632 (±846)^b^	1140 (±585)^a^	.006
CRP	5.67 (±5.57)^a^	4.87 (±5.13)^a^	5.80 (±6.59)^a^	6.89 (±5.31)^a^	.571	3.34 (±6.28)^a^	3.40 (±5.22)^a^	4.32 (±5.39)^a^	4.34 (±4.24)^a^	.772
S-protein	7.16 (±0.64)^a^	7.00 (±0.80)^a^	6.68 (±1.00)^a,b^	6.24 (±1.14)^b^	< .001	7.13 (±0.60)^a,b^	7.26 (±0.60)^b^	6.82 (±0.78)^a^	6.41 (±0.61)^c^	<0.001
S-albumin	3.75 (±0.56)^a^	3.72 (±0.66)^a^	3.44 (±0.70)^a,b^	3.10 (±0.68)^b^	< .001	4.15 (±0.62)^a^	4.16 (±0.58)^a^	3.74 (±0.75)^b^	3.36 (±0.45)^c^	<0.001

1) Statistical significances were tested by one-way analysis of variances among groups.

2) The same letters indicate nonsignificant difference between groups based on Scheffe multiple comparison tests.

Data are presented as mean ±SD.

WBC = white blood cell; Lymph-percent = percentage of lymphocyte; Abs-lymph-count = Absolute lymphocyte count; CRP = C-reactive protein; S-protein = serum protein; S-albumin = serum albumin

The number of patients with lymphopenia (defined as an absolute lymphocyte count <500) was 19 (9.9%) in the QFT-GIT group and 10 (4.7%) in the T-SPOT.*TB* group. Of these lymphopenia patients, 10 in the QFT-GIT group and 8 in the T-SPOT.*TB* group were immunocompromised.

### Factors influencing the sensitivity of IGRAs

Serum protein and albumin influenced the sensitivity of QFT-GIT according to age. Lymphopenia independently influenced the sensitivity of QFT-GIT (Table 5). In univariate analysis, the factors that influenced the sensitivity of T-SPOT.*TB* according to age were lymphopenia, serum protein (or serum albumin), CRP, and being immunocompromised (Table 6).

**Table 5:** Factors influencing the sensitivity of QFT-GIT according to age group.

	OR	95% CI	*p-value*
Age group*	0.596	0.394–0.901	0.014
Absolute lymphocyte count*	1.000	1.000–1.001	0.262
Age group^†^	0.617	0.401–0.950	0.028
Lymphopenia^†^	0.155	0.056–0.431	<0.001
Age group ^‡^	0.682	0.444–1.050	0.082
Serum protein^‡^	1.714	1.148–2.559	0.008
Age group ^§^	0.675	0.442–1.032	0.070
Serum albumin^§^	2.048	1.154–3.636	0.014
Age group ^∥^	0.534	0.346–0.825	0.005
CRP^∥^	0.954	0.893–1.019	0.165
Age group ^¶^	0.569	0.378–0.857	0.007
Immunocompromised^¶^	0.670	0.316–1.421	0.297
Age group **	0.555	0.371–0.829	0.004
Lesion of TB infection**	0.927	0.621–1.383	0.709
Age group^††^	0.572	0.383–0.855	0.006
Sex^††^	0.580	0.274–1.226	0.154
Age group^‡‡^	0.557	0.349–0.887	0.014
Absolute lymphocyte count^‡‡^	1.000	0.999–1.000	0.321
Lymphopenia^‡‡^	0.171	0.043–0.671	0.011
CRP^‡‡^	0.971	0.901–1.047	0.444
Immunocompromised^‡‡^	1.060	0.431–2.608	0.900
Lesion of TB infection^‡‡^	0.956	0.589–1.551	0.855
Sex^‡‡^	0.821	0.340–0.979	0.660

*, ^†, ‡, §, ∥, ¶, **, ††, ‡‡^ The factors with the same markers had undergone multivariate analysis with each other. Statistical significance was tested by binary logistic regression.

**Table 6:** Factors influencing the sensitivity of T-SPOT.*TB* according to age group.

	OR	95% CI	*p-value*
Age group*	0.582	0.341–0.993	0.047
Absolute lymphocyte count*	1.000	0.999–1.001	0.971
Age group ^†^	0.605	0.357–1.024	0.061
Lymphopenia^†^	0.559	0.104–3.014	0.499
Age group ^‡^	0.664	0.382–1.155	0.147
Serum protein^‡^	1.469	0.759–2.843	0.254
Age group ^§^	0.630	0.357–1.110	0.110
Serum albumin^§^	1.260	0.595–2.670	0.546
Age group ^∥^	0.519	0.263–1.025	0.059
CRP^∥^	0.952	0.858–1.057	0.356
Age group ^¶^	0.715	0.400–1.279	0.258
Immunocompromised^¶^	0.298	0.104–0.855	0.024
Age group **	0.571	0.338–0.963	0.036
Lesion of TB infection**	1.532	0.746–3.142	0.245
Age group ^††^	0.592	0.351–1.001	0.050
Sex^††^	3.094	1.063–9.005	0.038
Age group^‡‡^	0.593	0.344–1.024	0.061
Absolute lymphocyte count^‡‡^	1.000	0.999–1.001	0.978
Lesion of TB infection^‡‡^	1.393	0.672–2.884	0.373
Sex^‡‡^	2.906	0.989–8.544	0.052

*, ^†, ‡, §, ∥, ¶, **, ††, ‡‡^ The factors with the same markers had undergone multivariate analysis with each other. Statistical significance was tested by binary logistic regression

In multivariate analysis, the sensitivity of QFT-GIT according to age group still showed a significant decline when adjusted for absolute lymphocyte count, lymphopenia, CRP, being immunocompromised, location of TB lesion, sex (viz., the factors that did not influence the sensitivity of QFT-GIT according to age group, Table 5). In contrast, the decline in T.SPOT.*TB* sensitivity according to age was statistically insignificant when adjusted for absolute lymphocyte count, location of TB lesion, and sex (Table 6).

## Discussion

In summary, T-SPOT.TB showed higher overall sensitivity than QFT-GIT (91.0% vs. 80.2%). Both QFT-GIT and T-SPOT.*TB* showed statistically significant decreases in sensitivity according to age, and the degree of the decline was larger for QFT-GIT than for T-SPOT.*TB*. However, the sensitivity decline according to age of T-SPOT.*TB* but not QTF-GIT was statistically insignificant when adjusted for the factors that influence the sensitivity of IGRA.

There have been many contradictory reports on the relationship between IGRAs and age. Kobashi *et al*. [9, 11] reported that there was no significant difference in the positive rate for the QuantiFERON TB-2G test between elderly and younger patient groups. Kamiya *et al*. [10] also reported that the sensitivity of QFT-GIT was similar between elderly and young age groups; however, the false-positive rate was increased in elderly patients with comorbidities. However a recent study reported contradictory results. Jeon *et al*. [12] stated that inflammatory markers such as CRP and old age affected the false-negative rate of QFT-GIT. Kwon *et al*. [13] also reported that age 65 or over was related to false-negative QTF-GIT results. Our results are consistent with recent results showing that QTF-GIT test results were affected by old age. There are fewer studies on the relationship between age and T.SPOT.*TB* than on age and QFT-GIT. In the T-SPOT.*TB* assay, older age affected negative results [15, 22] or indeterminate results [23]. In contrast, Chee CB *et al*. (17) reported that the sensitivity of T-SPOT.*TB* did not differ significantly according to age. Another study reported that age did not affect indeterminate results for extrapulmonary TB [24]. Our results also showed that the sensitivity of the T-SPOT.*TB* assay was not affected by age even when adjusted for other factors.

For both IGRA tests, the absolute lymphocyte count and levels of serum protein and serum albumin were significantly lower in the elderly group than in the young age group. Lymphopenia, hypoalbuminemia, and immunosuppression have been reported to lead to negative and indeterminate IGRA results [25–28]. Diminished levels of total protein or albumin, which are indicators of malnutrition, can prevent a proper immune response [29].

In our study, the elderly group had the lowest mean absolute lymphocyte count (Table 4). It is well known that T-cell-mediated responses decrease with increasing age [6]. The T-SPOT.*TB* assay requires separation of PBMCs from heparinized whole blood before culturing PBMCs with TB antigens. The cells (250,000 per well) are incubated in a 96-well plate and exposed to TB antigens [30]. Therefore, T-SPOT.*TB* can be useful in patients with decreased lymphocyte counts [31]. It is possible that QFT-GIT is affected by both the number and function of lymphocytes. The fact that T-SPOT.*TB* is less affected by lymphocyte count due to the test characteristics (PBMC isolation, and addition of PBMCs to T-SPOT.*TB* 96-well plate [250,000/well]) may be the reason for the lesser decrease in sensitivity according to age relative to that of QFT-GIT.

In our study, the sensitivity of QFT-GIT according to age was significant when adjusted for immune deficiency, suggesting that the effect of age on the sensitivity of QFT-GIT was independent of immune status (Table 5). However, immune deficiency was a significant factor affecting the sensitivity of T-SPOT.TB, even after adjustment for age (Table 6). The sensitivity of T-SPOT.TB according to age was insignificant when adjusted for immune status. There have been many contradictory reports on the relationship between the sensitivity of T-SPOT.TB and immunosuppression [32–37].

In this study, there were a relatively high number of DM patients among the immunocompromised patients in the T-SPOT.TB group. Diabetes is known to be associated with lower levels of mycobacterial antigen-specific IFN-γ release [38]. However, another previous study revealed that T-SPOT.*TB* sensitivity was not affected by diabetes [39].

Although the T-SPOT.*TB* group had fewer study subjects with extrapulmonary TB, the sensitivity of this test was nevertheless higher than that of QFT-GIT (92.1% vs. 80.5%) for patients with extrapulmonary TB. Some studies have reported the diagnostic sensitivity of T-SPOT.*TB* in extrapulmonary TB in the range of 83.4% to 93.3% [24, 40, 41] and that it is higher than that of QFT-GIT [41]. Our results strengthen these reports.

This study was conducted in patients whose TB diagnosis had been confirmed by mycobacterial culture, histologic evidence, and/or TB PCR results. This enabled us to exclude the gray zone of diagnosis, which strengthened the study results. The total number of study patients (404) was larger than that of previous studies. Moreover, the IGRAs were conducted before initiation of anti-TB medications. The sensitivity results were similar to those of a previous meta-analysis [42]. However, our study has several limitations. First, it was not a head-to-head comparison of the two IGRA techniques. Furthermore, the QFT-GIT and T-SPOT.*TB* tests were conducted in different hospitals. Nevertheless, the number of subjects and the ratio of the sexes were not significantly different between the two hospitals, and the overall sensitivity was similar to that in previous reports. Second, the T-SPOT.*TB* group had relatively fewer subjects with extrapulmonary TB than the QFT-GIT group. As mentioned above, T-SPOT.*TB* showed high sensitivity in patients with extrapulmonary TB. Because our study included subjects thought to be definitely infected with TB, the test results may not be affected by the differences in the TB lesions. Third, because the design of this study is retrospective, we were unable to verify any history of BCG injection or smoking and analyze the impact of these factors on IGRA sensitivity according to age. Additionally, the full spectrum of laboratory tests had not been performed on <10 patients (except CRP). However, several results from patients who had complete laboratory data showed statistically strong significant differences between age groups.

## Conclusions

There was a sensitivity decline according to increasing age for both the QFT-GIT and T-SPOT.*TB* tests. However, the sensitivity decline of T-SPOT.*TB* according to age was statistically insignificant when adjusted for the factors that influence the sensitivity of T-SPOT.*TB* according to age. Our study strongly suggests that the results of QFT-GIT but not T-SPOT.*TB* should be interpreted cautiously in elderly patients.
